# Targeting M2 Macrophages with a Novel NADPH Oxidase Inhibitor

**DOI:** 10.3390/antiox12020440

**Published:** 2023-02-10

**Authors:** Sébastien Dilly, Miguel Romero, Stéphanie Solier, Olivier Feron, Chantal Dessy, Anny Slama Schwok

**Affiliations:** 1 Gustave Roussy Cancer Center, CNRS UMR 8200, F-94805 Villejuif, France; 2 Pole of Pharmacology and Therapeutics (FATH), Institute of Experimental and Clinical Research (IREC), Université Catholique de Louvain, B-1200 Brussels, Belgium; 3 Department of Pharmacology, School of Pharmacy, Center for Biomedical Research (CIBM), University of Granada, 18071 Granada, Spain; 4 Gustave Roussy Cancer Center, INSERM U1170, F-94805 Villejuif, France; 5 WELBIO Department, WEL Research Institute, Avenue Pasteur, 6, B-1300 Wavre, Belgium

**Keywords:** macrophage, ROS inhibition, vascular tone, NADPH oxidase, NADPH, molecular modeling, macrophage differentiation, tumor microenvironment

## Abstract

ROS in cancer cells play a key role in pathways regulating cell death, stemness maintenance, and metabolic reprogramming, all of which have been implicated in resistance to chemo/ immunotherapy. Adjusting ROS levels to reverse the resistance of cancer cells without impairing normal cell functions is a new therapeutic avenue. In this paper, we describe new inhibitors of NADPH oxidase (NOX), a key enzyme in many cells of the tumor microenvironment. The first inhibitor, called Nanoshutter-1, NS1, decreased the level of tumor-promoting “M2” macrophages differentiated from human blood monocytes. NS1 disrupted the active NADPH oxidase-2 (NOX2) complex at the membrane and in the mitochondria of the macrophages, as shown by confocal microscopy. As one of the characteristics of tumor invasion is hypoxia, we tested whether NS1 would affect vascular reactivity by reducing ROS or NO levels in wire and pressure myograph experiments on isolated blood vessels. The results show that NS1 vasodilated blood vessels and would likely reduce hypoxia. Finally, as both NOX2 and NOX4 are key proteins in tumors and their microenvironment, we investigated whether NS1 would probe these proteins differently. Models of NOX2 and NOX4 were generated by homology modeling, showing structural differences at their C-terminal NADPH site, in particular in their last Phe. Thus, the NADPH site presents an unexploited chemical space for addressing ligand specificity, which we exploited to design a novel NOX2-specific inhibitor targeting variable NOX2 residues. With the proper smart vehicle to target specific cells of the microenvironment as TAMs, NOX2-specific inhibitors could open the way to new precision therapies.

## 1. Introduction

Tumor cells interact with their surroundings to create an adequate microenvironment for their growth, in a similar manner to that observed in inflammation and in wound healing and tissue remodeling. The polarization of macrophages in the tumor-promoting “M2” type is a fundamental event in the establishment of the tumor microenvironment that supports drug resistance and down-regulates the immune response. Tumor-associated macrophages (TAMs) and tumor-associated neutrophils (TANs), are immune effector cells that are recruited to tumor tissue and release immunosuppressive cytokines, chemokines, and growth factors [1], which favor relapse and tumor growth. Clinical studies showed the association between a high density of TAMs often residing within tumor hypoxic compartments and poor clinical prognoses in cancer patients [2,3]. Tumor-associated neutrophils TANs, like macrophages, may acquire a pro-tumorigenic “N2” phenotype [4]. TANs are characterized by the up-regulation of several chemokines and are promoted by the immunosuppressive transforming growth factor β (TGF-β), itself preferentially produced under hypoxia.

Superoxide ions and more generally reactive oxygen species (ROS) play an important role in the pro-tumoral functions of TAMs and TANs [5,6]. More generally, a balance between ROS generating and ROS-detoxifying mechanisms is a precarious equilibrium in cancer cells. Accordingly, any strong deviation of the redox status of the tumor microenvironment may lead to potential alteration of cancer cell growth, as the tumor microenvironment might become inadequate to fulfil the high metabolic demand of tumor cells.

The main source of ROS in TAMs and TANs is the enzyme NADPH oxidase-2 (NOX2) found at the plasma membrane of phagocytic cells generating superoxide anions upon activation. Macrophage differentiation and the occurrence of “M2”-type TAMs during tumor development are impaired by NOX2 deficiency, reducing the secretion of cytokines and contributing to the inhibition of tumor growth and metastasis [7]. NOX2 proteins of phagocytic cells have also been linked to aerobic glycolysis [8].

Targeting NOX2 may thus represent an attractive strategy to alter the fitness of pro-tumorigenic immune cells. Interestingly, this approach may possibly also impact tumor perfusion and thereby reduce hypoxia which per se induces and maintains the phenotypes of TAMs and TANs. Indeed, while angiogenesis is usually associated with dysfunctional immature blood vessels in mice, it is now well accepted that a more mature blood vasculature can be pharmacologically modulated and reduce tumor hypoxia [9,10,11].

In this paper, we use an inhibitor of NADPH oxidase(s), targeted at their C-terminal dehydrogenase domain, called nanoshutter-1, NS1 [12,13] to impede the differentiation of tumor-associated “M2” macrophages from blood monocytes. We previously showed NS1 binding to recombinant NOX2 by exploiting NS1 fluorescence once bound to recombinant NOX2 [14]. NS1 inhibited NOX2 activity in an in vitro model [12,14] and in mice, as well as macrophages activated by PMA or triggered by Influenza A virus [14]. Moreover, NS1 decreased the level of ROS formed by endothelial HUVEC cells and by isolated mice aorta determined by EPR spin trapping [15]. Melanoma A375 cell growth was inhibited by treatment with NS1 [15], but this was not the case for breast cancer cells [12]. As the presence of TAMs in the microenvironment is a hallmark of many aggressive cancers and associated with resistance to treatment, impeding the differentiation of tumor-associated “M2” macrophages from monocytes could be a valuable strategy independent of the direct effect of the inhibitor on the tumor cells themselves.

Importantly, we also document here that NS1 leads to a significant vascular relaxation, thereby suggesting the capacity of such compound to reduce tumor hypoxia.

As both NOX2 and NOX4 are key proteins in tumors and their microenvironments, we investigated whether NS1 would differently probe these two proteins we modeled by homology modeling. As two structures of NOX2 obtained by cryo-EM, X-ray crystallography were recently published, we compare our models with the accessible data on the PDB and the Alpha fold site [16,17,18]. Differences within the C-terminal domains of NOX2 and NOX4 open the way for the design of a novel inhibitor specific to NOX2 as compared to NOX4.

## 2. Materials and Methods

### 2.1. Reagents

Mitotracker (red or far-red) and CellRox deep red were purchased from Thermo-Fischer and Invitrogen (Les Ulys, France), respectively; antibodies against NOX2 (ab80897) and p22^phox^ (ab75941) were acquired from Abcam; p47^phox^ (sc7660) and p67^phox^ (sc7663) were acquired from Santa Cruz; and NS1 was synthesized by Innoverda according to the published synthesis [19].

### 2.2. Isolation of Blood Monocytes and Differentiation of Blood Monocytes by CSF1

Peripheral blood samples were collected from healthy donors by the Etablissement Français du Sang. CD14^+^ monocytes were sorted using microbeads according to the manufacturer’s instructions (Miltenyi Biotec, Somerville, MA, USA), cultured in RPMI 1640 with glutamine (Thermo Fisher Scientific, Les Ulys, France) supplemented with 10% fetal bovine serum, and exposed to 100 ng/mL CSF-1 (R&D Systems, Minneapolis, MN, USA) to generate their differentiation, in the absence or presence of 15 μM NS1 or 500 μM Tiron (Santa Cruz, Dallas, TX, USA).

### 2.3. Flow Cytometry Analysis of Human Macrophages

To explore their phenotypes, human macrophages were washed with ice-cold PBS, incubated with Fc block (Human TruStain FcX, Biolegend, London, UK, 1/20 dilution) for 15 min, incubated with the antibodies for 20 min at 4 °C, washed, then the fluorescence was measured with a BD LSRFortessa X-20. The antibodies used were the following: APC/Alexa750-CD71 (#A89313, Beckman coulter, Brea, CA, USA), Krome orange-CD14 (#B01175, Beckman coulter), Pacific Blue-CD16 (#A82792, Beckman coulter), and PE-CD163 (#556018, BD). The data were analyzed with FlowJo software v. 10.0.00003.

### 2.4. Cell Morphology

Phase contrast images were captured with a Nikon Eclipse TE300 microscope before manually tracing the long and short axes of each cell (long axis, longest length of the cell; short axis, length across the nucleus in a direction perpendicular to the long axis) to measure the elongation factor as the ratio of these axes [20].

### 2.5. Phagocytosis

We used a gentamicin protection assay. CSF-1-treated monocytes (0.25 million) were infected with ampicillin-resistant E. coli K12 (MOI = 300) (Thermo Fisher Scientific) for 20 min in RPMI 1640 (Thermo Fisher Scientific) supplemented with 10% fetal bovine serum. Then, the cells were washed three times, incubated with RPMI 1640 supplemented with 10% fetal bovine serum and gentamicin (50 μg/mL) (Thermo Fisher Scientific) and lysed in PBS with 0.1% Triton X-100. The bacteria that penetrated the cells were released by Triton treatment and plated on LB plates containing ampicillin (100 μg/mL). Colonies were counted on the next day, allowing us to calculate the number of bacteria ingested by the macrophages.

### 2.6. Cytokine Profile in Human Macrophages Supernatants

IL-1 beta, IL-4, IL-6, and IL-13 concentrations were measured using the human Pro-Inflammatory Combo 1 U-Plex (MSD, Rockville, MD, USA) in cell culture supernatant. Chemiluminescence signal was measured on a Sector Imager 2400 (MSD).

### 2.7. Confocal Imaging

#### 2.7.1. Immunofluorescence of Living Cells

Confocal images were acquired on a Leica SPE Confocal system, sequential acquisition using 63 X/1.3 oil immersion objective. The 3D pictures were realized with the Bitplane Imaris software v. 7.7.2.

Macrophages are adherent cells which need to be gently detached from the flask. The suspension was centrifuged at 1500 rpm for 5 min and suspended in 1 mL saline and counted to obtain 200,000 cells in 400 µL medium. CellROX deep red (5 µM) and red mitotracker (diluted to 1/10,000) were added to the cells in the absence or presence of NS1 (10 µM) and incubated 30 min at 37 °C. The cells were rinsed twice with saline to remove the medium. We used red or far red fluorochromes to avoid overlap with the broad fluorescence spectrum of (NOX-bound) NS1 [14].

#### 2.7.2. Experiment in Fixed Cells

Cells were washed in PBS, fixed with 4% formaldehyde in PBS for 20 min, washed with PBS, post-fixed and permeabilized with cold (−20 °C) 70% ethanol for 20 min, washed with PBS, blocked with 8% bovine serum albumin (BSA) in PBS for 1 h, washed with PBS, incubated with the first antibody in 1% BSA in PBS for 2 h, washed with PBS, incubated with secondary antibody conjugated with adequate Alexa red or deep red markers for 1 h, washed with PBS, and mounted by using Vectashield mounting medium with DAPI (Vector Laboratories, Burlingame, CA, USA). To stain the mitochondria, mitotracker red (Thermo Fisher Scientific, 100 nM) was incubated 30 min with the living cells at 37 °C.

### 2.8. Cell Viability Tests

Cells derived from the thoracic aorta of embryonic rats (A7r5, Sigma, Saint Quentin Fallavier, France) were incubated at 37 °C for 2 h in the presence of serial dilutions of NS1 (1 μM–1 mM). The cell supernatants were removed and replaced with fresh medium before MTT analysis, as previously described [15]. Briefly, at the chosen time, 20 μL of 5 mg/mL 3-[4,5-dimethylthiazol-2-yl]-2,5-diphenyl tetrazolium bromide (MTT) in PBS was added to the cells and further incubated at 37 °C. After washing, 100 μL of DMSO was added to each well, and absorbance at 570 nm was measured using a multi-well Plate Reader (Model VICTORTM X4, PerkinElmer, Villebon-sur-Yvette, France) with a subtraction of blank value at 630 nm. The results were expressed as percentage of viability ± SEM versus control condition from four experiments. Significant differences between groups were calculated by ANOVA followed by a Dunnett post-hoc test.

Raw macrophage cell viability in the presence of increasing concentrations of NS1 (10–200 μM) was tested by staining with crystal violet. MTT tests were also performed in the same concentration range.

### 2.9. Modeling

A model of NOX2 dehydrogenase domain was previously built based on the X-ray structure of the C-terminal region (spanning the amino acid residues 385 to 570) of gp91 protein that was available from the Protein Data Bank (PDB) (PDB id 3A1F) and homology modeling of CsNOX5 [12,14,21]. The resulting model was then optimized by the molecular dynamics simulation of 100 ns using the GROMACS 2018 package [22]. The structure with the lowest energy during the last 10 ns was selected for the docking study.

NS169 was designed from NS1, based on the principles that its biphenyl-like chromophore should be shorter that the chromophore of NS1, with minimal rotation around the C-C bond, restrained by a substitution of two OH groups on the phenyl ring and a short positively charged amine carried by a rigid group to catch the NOX2-specific E540. Once docked into NOX2 model by flexible docking using the flexible docking protocol of Discovery Studio v18, the resulting complex was then submitted to a 30 ns molecular dynamics simulation using the GROMACS 2018 package in duplicate. A clustering (RMSD cutoff: 2 Å) was finally applied to extract the most (populated) representative structure.

### 2.10. Animals and Experimental Protocols

Twelve-week-old C57BL/6J male mice were obtained from Janvier Labs (Le-Genest-Saint-Isle, France) and housed in a temperature-controlled room with a 12:12 light-dark cycle and food and water ad libitum.

All experimental procedures and protocols were approved by the local Ethics Committee “Comité d’Ethique pour l’Expériementation animale”, Secteur des Sciences de la Santé, Université Catholique de Louvain (agreement UCL/MD/2007/010 and 2012/MD/UCL/004), according to National Care Regulations and Directive 2010/63/EU of the European Parliament and of the Council.

### 2.11. Vascular Reactivity Studies on Pressure Myograph

Twelve-week-old C57BL/6J male mice were sacrificed; thoracic aortic rings and second branch mesenteric arteries were, respectively, mounted on a wire- or pressure-myograph as previously described [23]. Briefly, isolated mesenteric micro-arteries were mounted on a pressure myograph (Ionoptix, Amsterdam, The Netherlands). Vessels were left to recover for 45–60 min in no-flow conditions (40 mm Hg, 37 °C) in a calcium-containing physiological salt solution buffer (PSS). The vasomodulating properties of NS1 were evaluated on vessels constricted with KCl (50 mM) or phenylephrine (Phe 10 µM), before or after incubation to NS1 10 μM for 2 h. Changes in the external diameters (contraction) were tracked and measured with the Myoview software and expressed as percentages of the initial diameter (Mean ± SEM from 3 experiments). Paired t-tests were used to test for significance. Aortic rings (<2 mm in length) were mounted in a wire myograph (model 610M, Danish Myo Technology, Aarhus, Denmark) for isometric tension measurement as previously described [24]. After normalization of the internal diameter, arterial rings were left to recover for 45–60 min. The contractile response to high KCl solution (50 mM) in presence of indomethacin (100 μM) was evaluated in vessels with or without endothelium. NS1 solvent (PSS/ Control) or NS1 (10 μM) was added in the bath for 90 min after reaching the maximum concentration at KCl.

Results are expressed as percent of the contractile response to KCl at the moment of time of solvent/NS1 addition. Results are mean ± SEM of three to five independent experiments. Significant differences between groups were calculated by a two-way Anova.

## 3. Results

### 3.1. NS1 Inhibition of “M2” Macrophage Differentiation

**Figure 1:**
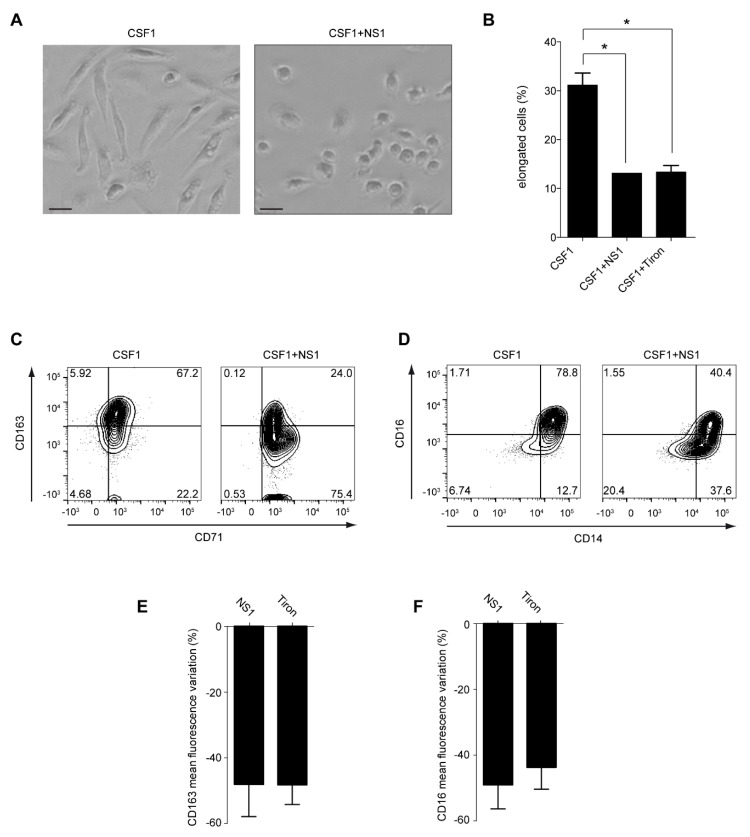
**NS1 modifies macrophage differentiation**. Monocytes from healthy donors were treated with CSF-1 (100 ng/mL, 3 days) in the absence or presence of NS1 (15 µM) or Tiron (500 μM). (**A**): Human CD14^+^ monocytes treated with CSF1 become adherent and elongated (left panel), a phenotype not observed after co-treatment with NS1 (right panel, scale 20 µm). (**B**): The fraction of cells with an elongated shape (elongation factor > 2.5) was measured in monocytes treated with CSF1, CSF1 + NS1, CSF1 + Tiron. Fisher test with Bonferroni correction. *: *p* ≤ 5 × 10^−2^ (**C**,**D**): Cytometric analysis of surface markers CD71 and CD163 (**C**) or CD14 and CD16 (**D**) of monocytes + CSF1 ± NS1. (**E**,**F**): Variation in the Mean fluorescence index of cell surface CD163 (**E**) and CD16 (**F**) expression in monocytes treated with CSF1, CSF1+ NS1, CSF1 + Tiron compared to monocytes treated with CSF1.

**Figure 2:**
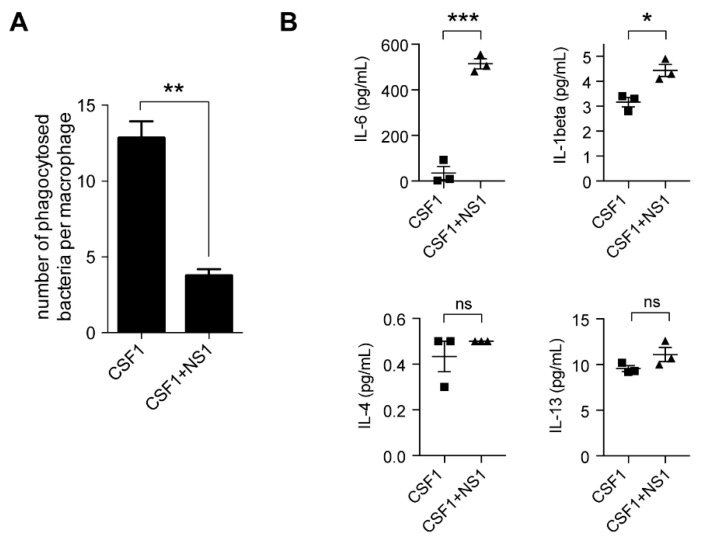
**NS1 changes the bacterial uptake and the cytokinic profile of CSF1-treated monocytes.** (**A**): Bacterial uptake was measured by the gentamicin protection assay in monocytes treated with 100 ng/ mL CSF1 alone or CSF1+NS1 (15 µM) during 3 days.. Mann–Whitney test. **: *p* < 10^−2^; (**B**): Inflammatory cytokines were measured in culture supernatants of human monocytes treated by CSF1 or CSF1+ NS1 at day 3. Results were expressed in pg/mL (ns, non-significant; *: *p* < 5 × 10^−2^, ***: *p* < 10^−3^ unpaired t-test).

Human blood monocytes isolated from healthy donors can be differentiated by the Colony-stimulated factor-1 (CSF1) into macrophages that presented a characteristic morphology. Indeed, the monocytes were round cells that did not attach to the surface, whereas the differentiated macrophages were adherent and acquired an elongated phenotype (Figure 1A,B). They were CD14^+^, CD16^+^, and CD163^+^ (Figure 1C,D), consistent with an “M2” phenotype. Moreover, they exhibited a phagocytic activity (Figure 2A). Treatment of the monocytes with NS1 inhibited the CSF1-induced macrophage differentiation as shown by the round phenotype and the decrease of the CD163 and CD16 markers, which is in agreement with the inhibition found with the superoxide scavenger, Tiron (Figure 1A–F). The decrease of the “M2” markers CD163 and CD16 induced by NS1 treatment was also seen in CSF1-treated monocytes of patients with a defective NOX2 (chronic granulomatous disease CGD patients) as shown by Solier et al. [25], linking these effects to NOX2 inhibition by NS1.

Additionally, the phagocytic capacity decreased in NS1-treated cells (Figure 2A). NS1 inhibited the CSF-1-induced macrophage differentiation by the inhibition of ROS formed by NOX2 (see below).

“M1” and “M2” macrophages are known to differ by their secretory repertoires, in particular cytokines and chemokines, whereas monocytes do not release these effectors. Figure 2B shows how NS1 modified the secretion of cytokines by CSF1-treated monocytes; the levels of the pro-inflammatory cytokines IL-6 and IL-1β, characteristic of a “M1”-like phenotype, were significantly enhanced by NS1. In contrast, IL-13, which is anti-inflammatory, was not significantly changed. The increase in pro-inflammatory cytokines suggests that NS1 likely shifted the macrophage phenotype toward an “M1” state, pointing out the plasticity of the macrophage phenotype as a function of their environment.

**Figure 3:**
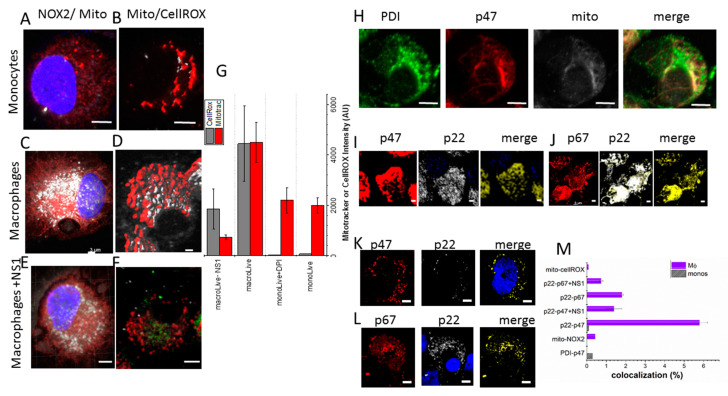
**Confocal imaging of living cells (Left A**–**F)** (**A**,**B**): monocytes isolated from blood of healthy donors; the red signal is the NOX2 and mitotracker in (**A**,**B**), respectively, the grey signal corresponds to the mitotracker and CellRox deep red reagent in (**A**,**B**), respectively; (**C**,**D**) Monocyte-derived macrophages (with CSF1-without NS1 treatment) using the same markers used for the living monocytes in (**A**,**B**); (**E**,**F**) the macrophages were treated with NS1 after their CSF1-induced differentiation from monocytes using the same markers as in (**A**,**B**); the green signal in (**F**) is the fluorescence of NS1; the ROS levels are quantified by the cell-permeable CellROX deep red reagent shown in light grey, the mitochondria are monitored by the red mitotracker reagent as quantified in (**G**); **Co-localization experiments in fixed cells (Right H**–**L)** The images (**H**–**L**) present co-localization experiments in fixed monocytes or CSF1-treated monocytes differentiated into macrophages**.** Each antibody is mentioned above each image, the merge, representing the colocalization is shown in yellow. The intensity of the colocalization is quantified in (**M**). We tested colocalization between partners of the activated NOX2 complex in “M2” macrophages formed by membrane bound NOX2 and p22^phox^ with p67^phox^ and p47^phox^ (p40^phox^ and rac1 are not labeled). It is known that p67^phox^ and p47^phox^ are located in the cytoplasm of the resting cells [23] in agreement with the images shown in (**H**); (**I**,**J**) are p22^phox^-p47^phox^ and p22^phox^-p67^phox^ co-localization signals in macrophages without NS1, respectively; (**K**,**L**) are p22^phox^-p47^phox^ and p22^phox^-p67^phox^ co-localizations with NS1, respectively. Scale bars are 3 µm. Quantification of the 3D images is shown in (**M**) and was performed with Imaris 7.3 software (http://www.bitplane.com/imaris, accessed on 6 February 2023).

To further investigate whether the phenotype of the already differentiated “M2” macrophages by CSF1 could be modified by applying NS1 after the differentiation, we imaged living monocytes and derived CSF1-treated monocytes with or without NS1 treatment as shown in Figure 3 using confocal microscopy. The living cells were co-labeled with the mitotracker reagent, shown in grey, and with a NOX2 antibody targeting NOX2 C-terminal, shown in red (Figure 3A,C,E). In another series of experiments, the ROS levels were monitored by the CellRox deep red reagent, shown in grey, and the mitochondria were labeled in red (Figure 3B,D,F).

The CellROX signal was low in monocytes, in agreement with low ROS production in these cells (Figure 3B). Consistently, the addition of diphenyliodonium, DPI, a general flavin inhibitor, only weakly inhibited the low ROS signal detected in monocytes (Supplementary Figure S1A).

In “M2”-macrophages, the CellRox level was consistent with high ROS levels, supporting higher mitochondrial activity in these cells (Figure 3C,D,G). Importantly, NS1 inhibited the CellROX signal by 2.4-fold in macrophages (Figure 3F,G) and also decreased the mitochondrial density as measured by the red mitotracker reagent (Figure 3E,F).

To further understand the mechanism of the NS1 inhibition of “M2” macrophages differentiation, we tested whether NS1 inhibited the active NOX2 complex in these cells. An active NOX2 complex requires the recruitment at the membrane of soluble factors, in particular p67^phox^ activator and p47^phox^ adaptor proteins, while p22^phox^ is inserted at the membrane interacting with NOX2.

In monocytes, where the NOX2 complex is mainly inactive, one expects a cytosolic localization of p67^phox^ as shown in Figure 3H by the partial co-localization of p47^phox^ with the ER-resident protein disulfide isomerase PDI and the mitotracker. Accordingly, only partial p22^phox^-p47^phox^ co-localization signals were observed in monocytes (Supplementary Figure S1B), which decreased in NS1-treated monocytes (Supplementary Figure S1C).

In CSF1-treated monocytes (“M2” macrophages), the NOX2 complex was active and generates ROS (Figure 3D), p22^phox^ and p67^phox^ should be co-localized; the same holds true for p22^phox^ and p47^phox^. The large co-localization signals shown in yellow in Figure 3I–J between p22^phox^ and p47^phox^ and between p22^phox^ and p67^phox^ were only observed in macrophages. Additionally, NOX2 staining partly overlapped with the mitotracker staining, suggesting that some NOX2 likely was localized at the mitochondria in “M2” macrophages (Figure 3C). The p47^phox^ recruited at the active NOX2 complex did not co-localize with the ER-resident, PDI.

NS1 disrupted the active NOX2 complex required for “M2” macrophage differentiation as shown by the large inhibition of co-localization signals and the large decrease of the CellRox signal [8,23] (Figure 3K,L,M,F, respectively).

These experiments strongly suggest that NOX2 inhibition by NS1 largely reduced the “M2” phenotype and would likely decrease the extent of infiltrated TAM in tumors. NS1 may also induce modifications of their metabolism, further suggested by the decrease of MTT signal with no decrease in macrophage cell viability determined by crystal violet staining (Supplementary Figure S2).

### 3.2. Effect of NS1 on Vascular Tone and Metabolism of Smooth Muscle Cells

**Figure 4:**
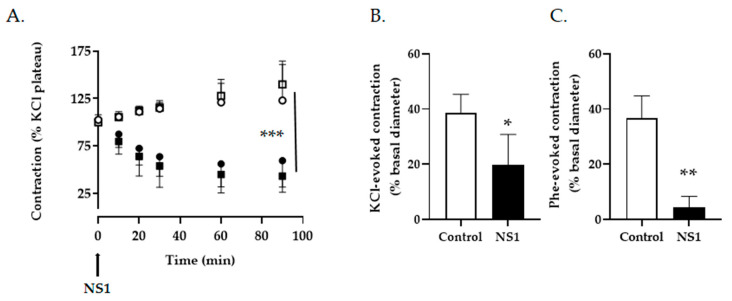
**NS1 inhibition of vascular contraction.** (**A**) Mice aortic rings with (circles) or without endothelium (squares) were pre-constricted in the presence of high KCl solution (50 mM) then exposed to 10 µM NS1 (black symbols) or solvent (open symbols) at the plateau phase of the contraction. Vascular tone was measured during 90 min after addition of NS1 and expressed as percent of the contractile response at time 0. Mean ± SEM of three to five independent experiments, two-way anova, *** *p* < 0.001. (**B**,**C**) Micro-mesenteric arteries (ext. diam: 140 ± 15 µm) were contracted in the presence of high KCl (50 mM) or Phenylephrine (10 µM) before and after incubation with NS1 (10 µM) for 2 h. Mean ± SEM of 3 independent experiments, paired t-test, * *p* < 0.05, ** *p* < 0.01.

To verify that, by blocking NOX or other NADPH-dependent enzymes, NS1 would not impede tissue perfusion, the contractile response of mice conductance and resistance vessels to NS1 was measured ex vivo (Figure 4). When added on pre-constricted aortic rings, NS1 promoted a significant relaxation that was sustained over time. A relaxation of similar amplitude was observed in vessels which have been deprived of their endothelium; consistent with a vasodilating process independent of endothelial nitric oxide synthesis (Figure 4A). More importantly, the relaxing properties of NS1 were also observed in the micro-circulation, as both KCl-induced or phenylephrine-induced contraction were significantly hampered after incubation of mesenteric micro-arteries in the presence of NS1 (Figure 4B,C).

**Figure 5:**
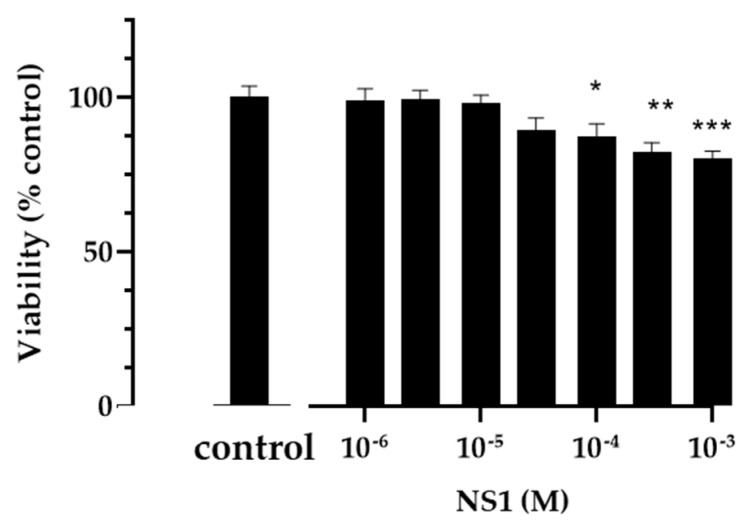
**Smooth muscle cell viability.** A7r5 smooth muscle cells were incubated for 2 h in the presence of various concentrations of NS1 (1 µM to 1 mM). Viability was estimated with a MTT assay. Results are expressed as percentage of viable cells in comparison to untreated cells (control), results are the mean +/− SEM of 4 experiments. * *p* < 0.05; ** *p* < 0.01; *** *p* < 0.001) 1-way Anova.

MTT assays were performed to ascertain that the relaxation process did not result from smooth muscle cell death or altered energetic metabolism. When incubated for up to 2 h, NS1 only minimally affected A7r5 cell viability (Figure 5). Together, this suggests that NS1 would not hinder tissue perfusion and oxygenation.

### 3.3. NADPH Sites of NOX2 and NOX4; Exploiting Their Variability to Design a NOX2-Specific Inhibitor

**Figure 6:**
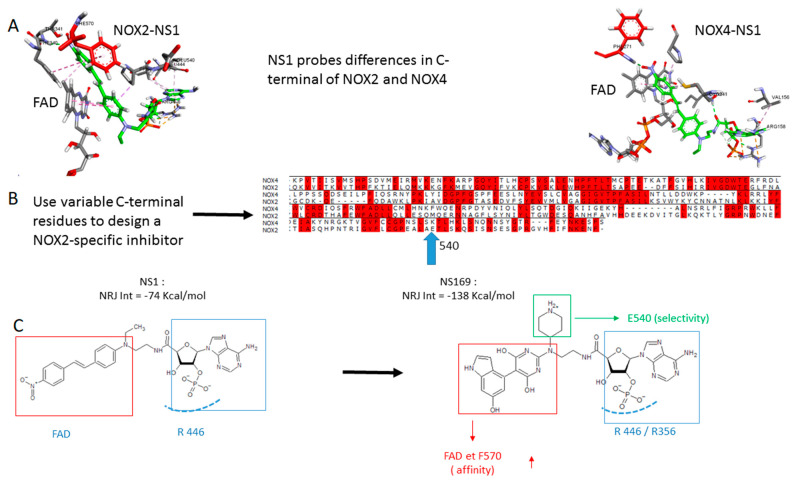
**From a pan-NOX inhibitor NS1 to a NOX2-specific inhibitor NS169.** (**A**) Comparison of the interactions of NS1 shown in green with NOX2 and NOX4; the C-terminal residue F570 in NOX2 is highlighted in red; (**B**) alignments of NOX2 and NOX4 C-terminal, identical residues are in red and variable residues in white; (**C**) Structures of NS1 and its NOX2-specific analog. The part of the compounds recognizing the conserved arginines/lysines of the protein is boxed in blue, the aromatic chromophore moieties recognizing FAD are boxed in red.

Taken together, it makes sense to develop a NOX2-specific inhibitor to target the microenvironment of tumors highly infiltrated with TAMs and reduce hypoxia. The question is, is it possible to exploit the C-terminal NADPH site of NOXes to perform this task? The design principles we developed for generating NS1 were based on the general recognition motif of NADPH via its 2′ phosphate group by the conserved arginine(s) of the protein of its C-terminal domain [12,13,14,15,19]. Therefore, if the C-terminal NADPH sites of NOX isoform were globally identical, NS1 should not discriminate between NOX isoforms. As a proof of concept, we generated models of the C-terminal domains of NOX2 and NOX4 using homology modeling, based on the structure of NOX5 [21] and the partial structure of NOX2 (PDB 3A1F) (Figure 6A and Figure S3). Two main differences are immediately visible: first, that the last residue F570 at NOX2 C-terminal (shown in red) stacks on NS1 (shown in green), whereas the same F points away from NS1 and FAD in NOX4. F570 is likely to be a regulatory residue restricting access to FAD, thus regulating electron transfer in NOX2. Such a process has been postulated by Magnani et al. in NOX5 and shown in NOS [21,26,27,28,29]. This is in agreement with NOX2 requiring activation to generate superoxide ions while NOX4 is always active, mostly regulated at the post-traductional level. The second (possibly related) difference is the interaction of NS1 with FAD which differs in NOX2 and NOX4, the effective insertion of NS1 within that pocket being larger in NOX4 than in NOX2. Thus, NS1 probes structural differences at the level of NOX2 and NOX4 C-terminal although the interaction energies of NS1 bound to NOX2 or to NOX4 are similar, being largely dependent on electrostatic interactions of NS1 2′ phosphate with the proteins arginine(s) (Table 1).

**Figure 7:**
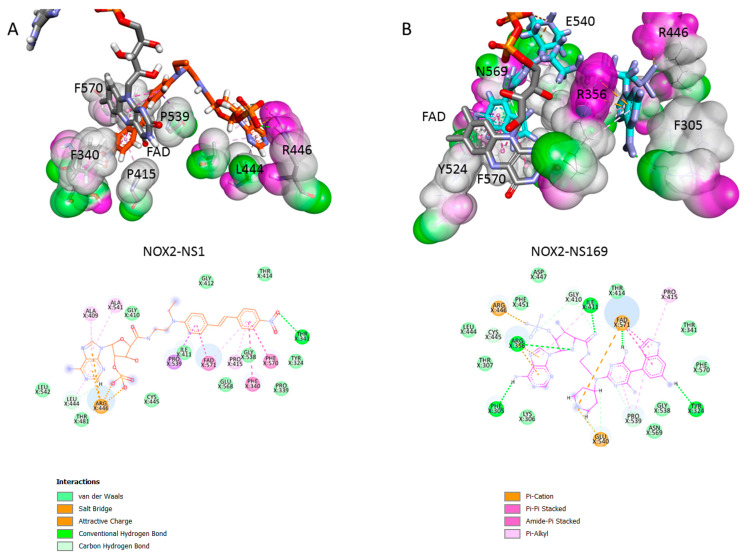
**Comparison of the interactions of NS1 (A) shown in orange and NS169 (B) shown in blue with NOX2:** FAD isoalloxazine is shown in grey sticks, its pyrophosphate groups in yellow (P) and red (O), the van der Waals surface is shown as H-bond donor or acceptor in violet and in green. Below are the 2D interaction maps between the inhibitors and interacting residues of NOX2, each residue being depicted according to the following color codes.

To design a NOX2- directed inhibitor, called NS169, we (i) strengthen its interactions with FAD and (ii) create new, stable interactions, with E540 identified as a NOX2 variable residue, largely differing from the corresponding lysine in NOX4 (Figure 6B). To achieve (i), we both shorten the chromophore by removing the double bond found in NS1 and replace the phenyl ring of NS1 by an extended purine ring for a better stacking with FAD (Figure 6C); (ii) to stably interact with E540 and P539, we restricted in NS169 the rotation around the single bond linking between the purine and the phenyl rings by substituting the phenyl ring with two OH groups. One OH group formed an H-bond with P539, while the other OH formed an H-bond with FAD pyrophosphate. This also enabled good interactions of the charged group of NS169 with E540 (Figure 7). When docked to NOX4, NS169 interactions with NOX4 were loose, consistent with the calculated interaction energies (Table 1 and Supplementary Figure S3). Taken together, despite the 2′ phosphate determinant being conserved in NADPH-binding proteins, the NADPH binding site is still adjusted for/ fitted to each protein regulation mode, which opens a chemical space we exploited for introducing a NOX2 specificity within the ligand binding to this site.

## 4. Discussion and Conclusions

The tumor microenvironment is associated with an immune-permissive environment and relapse. TAMs in multiple myeloma were negatively correlated with survival [30]. Inhibiting TAMs would also modify the dialog with cancer stem cells, as shown in pancreatic tumor where targeting TAM relieved immunosuppression, and improves chemotherapeutic responses [31]. Additional mechanisms of resistance to chemotherapeutic agents involve the induction of an ER stress and autophagy, blockade of the cell cycle, and induction of EMT [32].

### 4.1. NOX2 TAMs and NS1

To target the TAMs macrophages of the tumor microenvironment, it makes sense to inhibit NOX2, as TAMs rely on ROS mainly produced by NOX2 for proper differentiation [7]. Accordingly, the NS1 inhibition of ROS avoided monocytes differentiation into “M2” macrophages. Interestingly, the NS1 and CSF1 co-treatment of monocytes decreased the levels of the “M2” marker CD163 and CD16, similarly to the decrease of these markers observed in “M2” macrophages of chronic granulomatous disease (CGD) patients that harbor a dysfunctional NOX2 [25].

NS1 likely decreased the “M2” phenotype once formed by the treatment of monocytes with CSF1 since (i) it disrupted the activated NOX2 complex, and (ii) it increased the levels of pro-inflammatory cytokines IL6 and IL1β; thus NS1 possibly shifted the phenotype toward a “M1” phenotype. Additionally, the reduced level of the mitochondrial probe observed in the presence of NS1 together with the decrease of the MTT absorption relying on mitochondrial activity was consistent with a reported decrease of oxidative metabolism in “M1” versus “M2” macrophages [33]. We did not observe a change in macrophage cell viability determined by vital staining with crystal violet (Supplementary Figure S2). As the blockade of the cell cycle, the decrease of cell growth, and the decrease of STAT3 phosphorylation were previously shown with NS1 in metastatic A375 melanoma cells [13], it is likely that some of these events would take place in macrophages treated with NS1. Moreover, STAT3 is required for mitochondrial function, and the activated NOX2 complex we imaged in “M2” macrophage appeared to be at partly localized at the mitochondria, in addition with the localization at the membrane deduced from the colocalization of p22^phox^ and p67^phox^ or p47^phox^ (Figure 3I,J and Supplementary Figure S1D). NOX2 has also been localized in the ER compartment [34]. We could not find a co-localization of NOX2 with PDI in “M2” macrophages, but rather a partial co-localization in monocytes.

### 4.2. NOX Inhibition in Cancer and NOX2 Inhibitors

The interest of inhibiting NOX2 in cancer cells was shown by silencing or inhibiting NOX2 in breast cancer cell lines overexpressing IKKε and also in the development of resistance to tamoxifen [35]. Moreover, expression changes in both NOX2 and TRPM8 mRNA predicted poor clinical outcomes in estrogen receptor (ER)-negative breast cancer patients, linked to oncogenic KRas [36]. NOX2 in the context of KRas was also involved in the progression of myeloproliferative disease [37]. Additional evidence and the rationale of the interest of inhibiting NOX were reviewed in [38].

A number of NOX2 inhibitors were developed and shown to have interesting applications in the cancer field as reviewed in [39,40]. Recently, a redox-active Mn porphyrin was shown to scavenge ROS and decrease “M2” polarization, this inhibition being partly mediated through a decrease in STAT3 activation during IL4-induced “M2” polarization [5].

### 4.3. NOX Inhibition and Hypoxia

An important parameter contributing to the establishment and maintenance of the tumor microenvironment is hypoxia [41,42]. We have shown that, by decreasing ROS formed by NOX1/2 and NOX4 in melanoma cells, NS1 decreased lactate, a product of hypoxic metabolism, likely by inducing autophagy in these cells [13]. In this work, we probed the ability of NS1 to induce vasodilation in aortic rings and the microcirculation. NS1 was shown to inhibit ROS formed in aortic rings [15]. Here, we show further that NS1 induces a vasodilation in aorta and microvessels, a process independent of the presence of endothelium, ruling out an eNOS-dependent process and suggesting a NOX-dependent inhibition to enhance vasodilatation, in agreement with the effects of DPI and L-NAME on this process. In contrast, a NOX4 inhibitor would probably prevent H_2_O_2_-dependent relaxation [43]. Altogether, it is likely that enhanced vasodilation by NS1 would reduce hypoxia, but more work is needed to assess this hypothesis.

In conclusion, the pan-NOX inhibitor NS1 and the NOX2-specific inhibitor NS169 described in this work may be good mechanistic tools to understand the parameters switching from senescence to proliferation [44] and shed light on the mechanisms of aging and tumorigenesis. Associated with “smart vehicles” that would recognize specific features of TAMs or TANS, such inhibitors would be interesting candidates for precision therapies.

## Figures and Tables

**Table 1:** Comparison of NS1 binding to NOX2 and NOX4 with a selective NOX2 inhibitor.

Compound	R1	Chromophore	Protein	Interaction Energy Kcal/mol
NS1	Ethyl	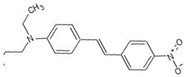	NOX2	−74
NS1	Ethyl		NOX4	−82
NS169	Hook C5H9NH2	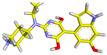	NOX2	−138
NS169	Hook		NOX4	−75

## Data Availability

The data presented in this study are available in the article and supplementary materials.

## Supplementary Materials

The following supporting information can be downloaded at: https://www.mdpi.com/article/10.3390/antiox12020440/s1, Supplementary Figure S1: Imaging of A living and B, C fixed monocytes and D of fixed “M2” macrophages. Supplementary Figure S2: Different dose responses of NS1 on Raw macrophages viability. Supplementary Figure S3: Comparison of the interactions of NS169 with NOX2 and NOX4.
