# Supplementary material for: Targeting M2 Macrophages with a Novel NADPH Oxidase Inhibitor
Source: Antioxidants (Basel). 2023 Feb 10;12(2):440. doi: 10.3390/antiox12020440 (PMC9951936; doi:10.3390/antiox12020440)
Supplement: Supplementary file 1 [file antioxidants-12-00440-s001.zip › antioxidants-2163388-supplementary.pdf]

antioxidants

Targeting M2 Macrophages with a Novel NADPH Oxidase Inhibitor

Sébastien Dilly [1,†], Miguel Romero [2,3,†], Stéphanie Solier [4,*,†], Olivier Feron [2,5], Chantal Dessy [2] and Anny Slama Schwok [1,*]

Supplementary Material

Supplementary Figure S1: Imaging of -A living and- B, C fixed monocytes and –D of fixed "M2" macrophages.

Supplementary Figure S2 : Different dose responses of NS1 on Raw macrophages viability

Supplementary Figure S3 : Comparison of the interactions of NS169 with NOX2 and NOX4:

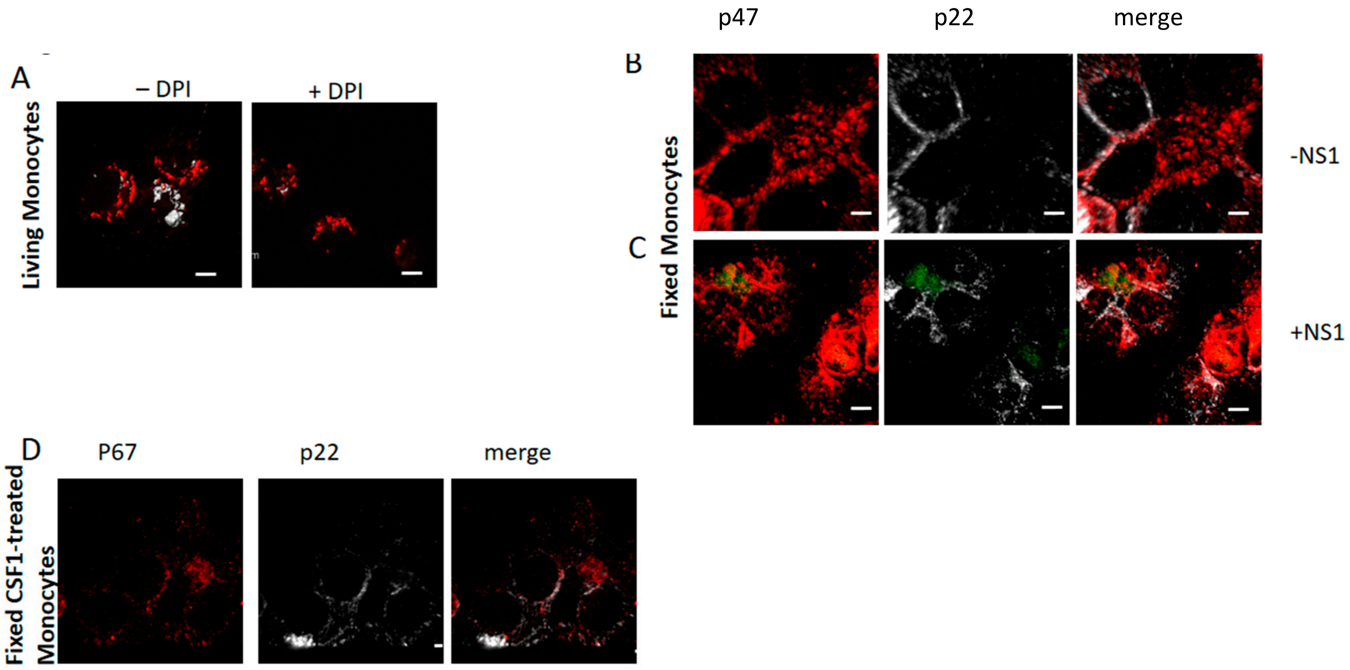

Supplementary Figure S1: Imaging of -A living and- B, C fixed monocytes and –D of fixed "M2" macrophages. A: Effect of the flavin inhibitor DPI on ROS levels shown in grey in living monocytes co-labeled with the red mitotracker; B and C show the co-labeling with p47 [phox] and p22[phox] in fixed monocytes without or with NS1 treatment; D: co-labeling of fixed "M2" macrophages with p67[phox] and p22[phox].

…

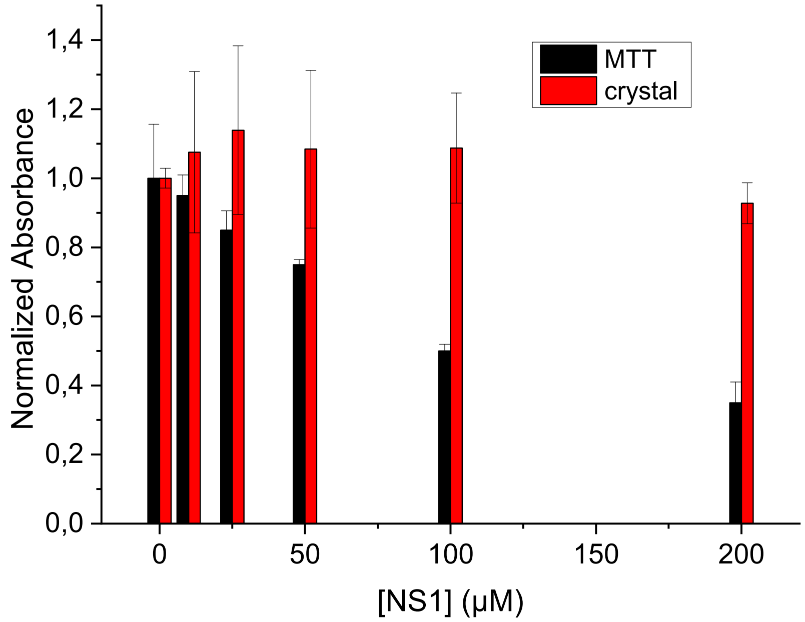

Supplementary Figure S2 : Different dose responses of NS1 on Raw macrophages : cell viability determined by crystal violet and MTT tests

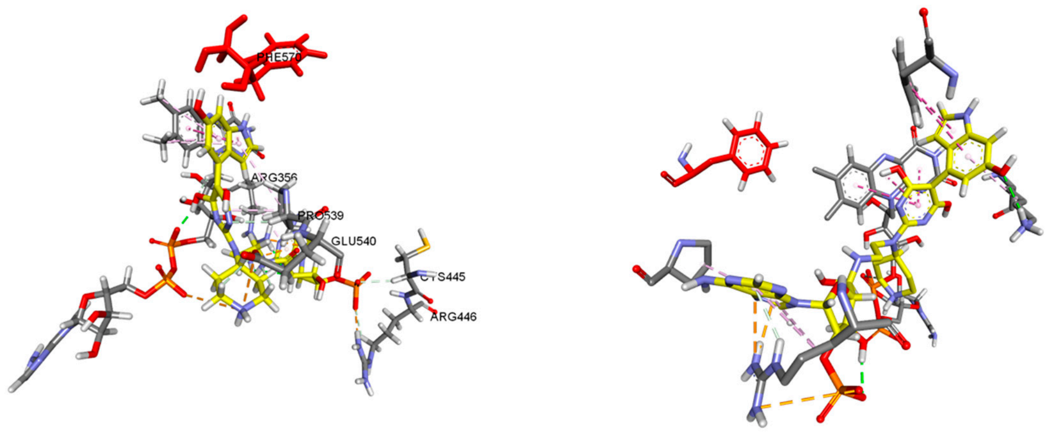

NOX2-NS169 NOX4-NS169

Supplementary Figure S3 : Comparison of the interactions of NS169 with NOX2 and NOX4:

Recognition by NS169 of NOX2-specific residues F305, E540 and N569 as compared to S270, K238 and a S267 in NOX4. The insert shows by comparison the partial stacking of NS1 with FAD whereas NS169 has a good stacking with FAD of NOX2, better shown in Figure 7.
